# Pressure controlled ventilation with volume guarantee improves outcomes in neonatal thoracoscopic esophageal atresia surgery

**DOI:** 10.3389/fped.2025.1524883

**Published:** 2025-05-19

**Authors:** Lv Kaimin, Luo Bijun, Luo Cheng, Wang Xiaoxia

**Affiliations:** Department of Anesthesiology, Maternal and Child Health Hospital, Nanning, Guangxi, China

**Keywords:** pressure controlled ventilation-volume guaranteed, neonates, thoracoscopic, esophageal atresia, one-lung ventilation, postoperative recovery

## Abstract

**Introduction:**

Neonatal thoracoscopic repair of esophageal atresia requires one-lung ventilation (OLV), which poses challenges due to immature lung development and low compliance, increasing risks of hypoxemia and barotrauma. While volume-controlled ventilation (VCV) ensures stable tidal volume, it may cause excessive airway pressures, whereas pressure-controlled ventilation (PCV) lacks volume guarantee. This study compared PCV with volume guarantee (PCV-VG) and conventional VCV to improve respiratory outcomes during OLV.

**Methods:**

A retrospective analysis was conducted on neonates (aged 1–7 days) undergoing thoracoscopic esophageal atresia repair with OLV. Patients were categorized into PCV-VG and VCV groups. Respiratory parameters (PaO_2_, PaCO_2_, airway pressures, dynamic compliance) were measured before, during, and after OLV. Propensity score matching (PSM) was used to balance baseline characteristics.

**Results:**

After PSM, 74 neonates (37 per group) were included. During OLV, the PCV-VG group exhibited significantly lower PaCO_2_, peak/mean airway pressures, and higher dynamic compliance compared to the VCV group (all *P* < 0.05). Postoperatively, PCV-VG was associated with shorter mechanical ventilation duration, ICU stay, and hospital stay (*P* < 0.05). Postoperative complication rates did not differ between groups (*P* > 0.05).

**Conclusion:**

PCV-VG offers superior ventilation parameters and faster recovery in neonatal thoracoscopic esophageal atresia repair, though it does not affect postoperative complication rates.

## Introduction

Esophageal atresia is a relatively rare congenital anomaly. Early diagnosis and timely surgical intervention are critical for improving prognosis. In recent years, the application of minimally invasive surgical techniques, such as thoracoscopic surgery, has potentially enhanced surgical outcomes and recovery in affected infants. Compared to open surgery, thoracoscopic procedures offer advantages such as reduced trauma and faster recovery, which may decrease the risk of postoperative complications (1). However, neonates face higher surgical and anesthetic risks due to immature lung development and low lung compliance, making them more susceptible to respiratory distress (2). The use of lung-protective ventilation strategies is one method to prevent pulmonary complications.

One-lung ventilation (OLV) is commonly employed during thoracoscopic surgery to provide a clear surgical field. Since OLV requires temporary closure of one lung for surgical manipulation, this significantly reduces the effective gas exchange surface, thereby increasing the risk of hypoxemia (3). To maintain adequate oxygenation, it is often necessary to increase the fraction of inspired oxygen and adjust ventilation parameters, such as increasing tidal volume (Vt) and respiratory rate (4). However, if these ventilation strategies are not precisely adjusted, they can lead to excessive airway pressures, subsequently increasing the risk of barotrauma (5, 6). High airway pressure can also result in severe complications such as pneumothorax and mediastinal emphysema (7).

There is currently no consensus on the optimal ventilation mode for OLV. Volume-controlled ventilation (VCV) ensures a stable tidal volume, but the associated high airway pressures may cause volutrauma or barotrauma. Pressure-controlled ventilation (PCV) can reduce airway pressures, but it carries the risk of lung injury due to alveolar overdistention. Pressure controlled ventilation-volume guaranteed (PCV-VG) combines the advantages of both PCV and VCV, allowing dynamic adjustment of airway pressure in response to shifts in lung compliance while maintaining the target tidal volume, thus reducing the risk of ventilator-associated complications (8). Previous studies have explored the use of PCV-VG in pediatric cardiac surgery and adult open-heart surgery with OLV, and some research has examined its effects on oxygenation and airway pressures (9, 10). However, studies on the application of PCV-VG in neonatal thoracoscopic surgery with OLV remain limited. Considering the unique physiological characteristics of neonates and the specific requirements of thoracoscopic surgery, this study investigates the application of PCV-VG in neonatal thoracoscopic procedures and evaluates its comparative effectiveness relative to VCV. The findings aim to contribute evidence-based insights for clinical decision-making in this context.

## Methods

### Patients

This retrospective study encompassed infants who received thoracoscopic surgery for esophageal atresia at the Maternal and Child Health Hospital in the Guangxi Zhuang Autonomous Region, spanning from January 2017 through March 2024. The criteria for inclusion were: age between 1 and 7 days and body weight ranging from 1.4 to 4.5 kg. All cases underwent OLV during surgery. Exclusion criteria included neonates with complex congenital heart disease or severe pulmonary hypoplasia or pneumonia. The participants were categorized into two distinct groups according to the mode of ventilation utilized during surgery: the VCV group and the PCV-VG group. The study protocol was reviewed and approved by the Ethics Committee of Maternal and Child Health Hospital of Guangxi Zhuang Autonomous Region [Approval Number: (2024)11-1]. As this study involved a retrospective analysis of cases and did not include personal information or privacy concerns, informed consent was waived.

### Ventilation protocol

Ventilation mode selection was guided by anesthesiologists’ clinical experience, intraoperative parameters such as peak airway pressure and dynamic compliance, equipment availability, and historical usage patterns. During the study period, VCV was predominantly used in early cases, while PCV-VG was progressively adopted with equipment upgrades and accumulated clinical experience.

### Data collection

Information was extracted from the electronic health records, encompassing details such as gender, gestational age, age post-birth, and the method of delivery, birth weight, American Society of Anesthesiologists (ASA) classification, type of esophageal atresia, and associated anomalies. ASA classification: ASA I indicates a healthy patient, ASA II a patient with mild systemic disease, ASA III a patient with severe systemic disease, ASA IV for life-threatening systemic disease, and ASA V for moribund patients.

Primary outcomes included PaO₂, PaCO₂, peak airway pressure (Ppeak), mean airway pressure (Pmean), plateau pressure (Pplat), and dynamic lung compliance (Cdyn). These parameters were documented at three specific intervals: 10 min prior to the OLV (T1), 30 min post the initiation of OLV (T2), and 10 min following the completion of OLV (T3).

Secondary outcomes included mean arterial pressure (MAP) and heart rate (HR) recorded before intubation (T0), at T1, T2, and T3. Additionally, the duration of surgery, time to extubation, ICU stay, length of hospital stay, and postoperative complications such as anastomotic leakage, anastomotic stricture, recurrent tracheoesophageal fistula, reflux esophagitis, and tracheomalacia were recorded.

### Statistical analysis

Analysis of the data was conducted utilizing SPSS or R software. Demographic details, preoperative status, and intraoperative metrics of the neonatal subjects were summarized using descriptive statistics. Findings were depicted as either the mean with standard deviation (mean ± SD) or the median within the interquartile range. Discrepancies were evaluated using t-tests or suitable non-parametric alternatives. Categorical data were represented in terms of counts and percentages, with differences assessed through the chi-square test or Fisher's exact test when necessary. In this study, propensity score matching (PSM) was employed to reduce confounding bias, with covariates including gender, gestational age, postnatal age, birth weight, mode of delivery, ASA grade, esophageal atresia type, and associated anomalies. The propensity scores were calculated using logistic regression, and 1:1 nearest neighbor matching was applied to match the groups. The matching process was implemented using the R package “MatchIt”. The statistical tests were two-sided, and a *P*-value of less than 0.05 was deemed to indicate statistical significance.

## Results

### Baseline characteristics of patients

**Figure 1:**
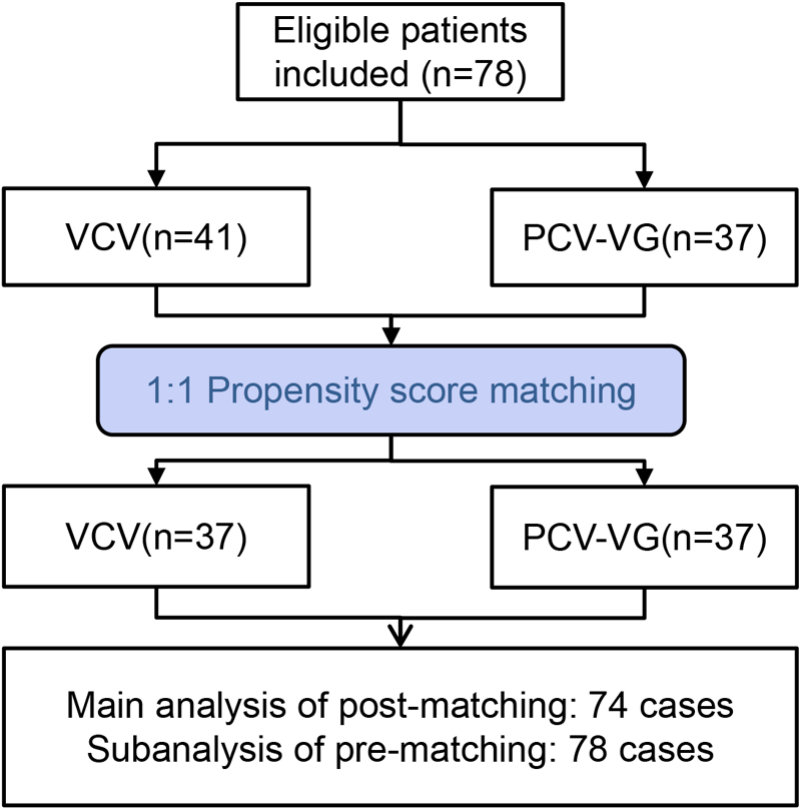
Flowchart of inclusion and grouping process for neonates undergoing thoracoscopic esophageal atresia surgery.

A total of 78 patients participated in the research, with the inclusion process outlined in Figure 1. After propensity score matching (PSM), 74 patients were finally included in the analysis, with 37 patients in each group. As shown in Table 1, there were no significant differences in gender, gestational age, postnatal age, method of delivery, birth weight, ASA classification, type of esophageal atresia associated anomalies and surgical duration between the two groups (*P* > 0.05).

**Table 1:** Baseline characteristics of patient.

Characteristics		VCV (*n* = 37)	PCV-VG (*n* = 37)	*P*
Gender *n* (%)	Male	15 (40.5%)	19 (51.4%)	0.484
	Female	22 (59.5%)	18 (48.6%)	
Gestational age [median (IQR)], weeks		38.0 (38.0, 39.0)	38.0 (37.0–39.0)	0.364
Postnatal age [median (IQR)], days		3.0 (2.0, 3.0)	2.0 (2.0–3.0)	0.612
Delivery *n* (%)	Vaginal	31 (83.8%)	32 (86.5%)	1.000
	Cesarean	6 (16.2%)	5 (13.5%)	
Birth weight [median (IQR)], kg		3.10 (2.80, 3.50)	3.10 (2.70–3.40)	0.522
ASA classification *n* (%)	Ⅲ	26 (70.3%)	26 (70.3%)	1.000
	Ⅳ	11 (29.7%)	11 (29.7%)	
Type of esophageal atresia *n* (%)	A	4 (10.8%)	4 (10.8%)	0.059
	B	0 (0%)	2 (5.4%)	
	C	33 (89.2%)	25 (67.6%)	
	D	0 (0%)	3 (8.1%)	
	E	0 (0%)	3 (8.1%)	
Associated Anomalies *n* (%)	No	14 (37.8%)	21 (56.8%)	0.162
	Yes	23 (62.2%)	16 (43.2%)	
Surgical duration [median (IQR)], min		141.0 (118.0, 155.0)	140.0 (119.0–158.0)	0.905

### Comparison of clinical outcomes

**Figure 2:**
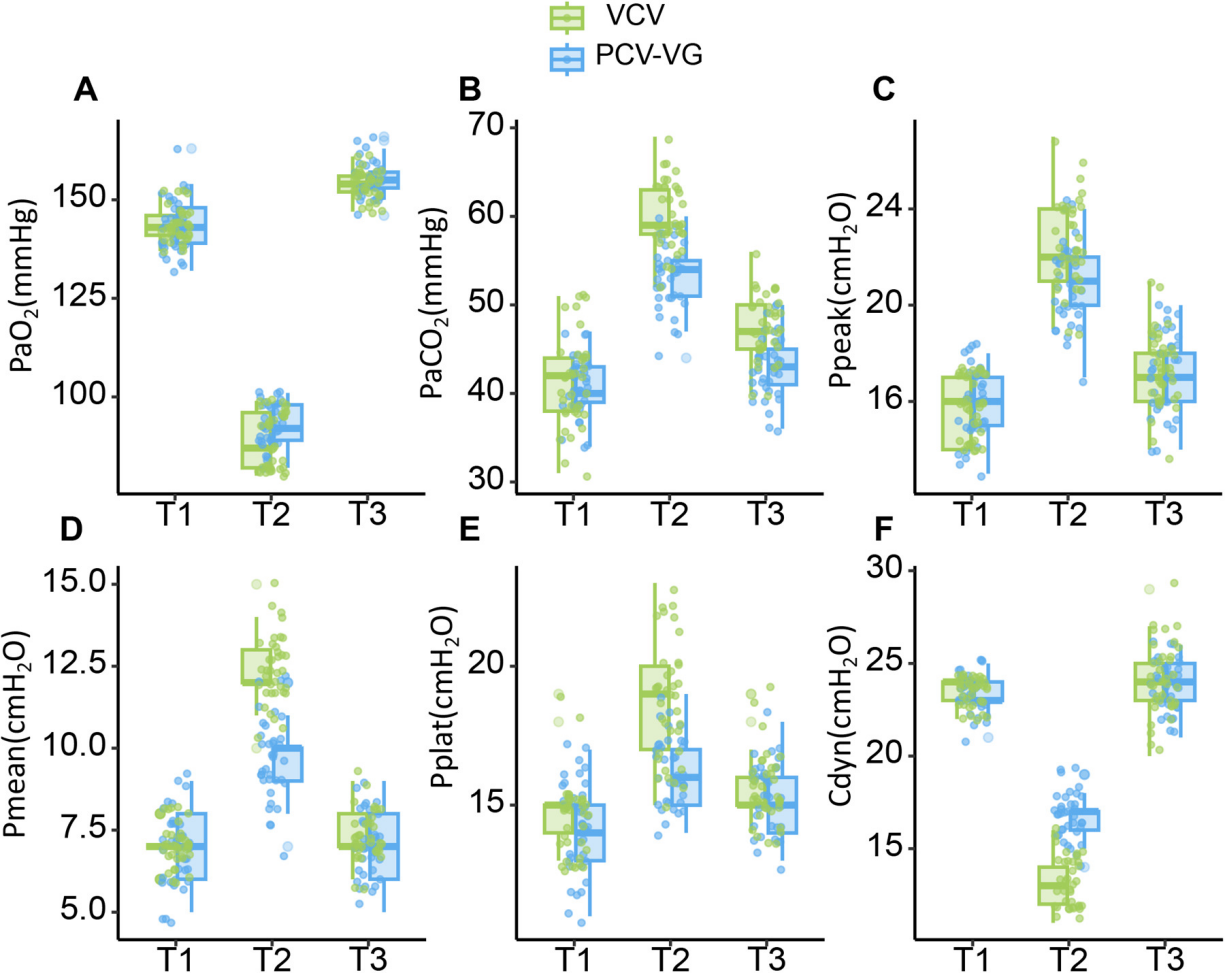
Changes in gas exchange function and respiratory mechanics. T1: 10 min prior to the OLV; T2: 30 min post the initiation of OLV; T3: 10 min following the completion of OLV. **(A)** The ordinate represents arterial oxygen partial pressure (PaO_2_, mmHg). **(B)** The ordinate represents arterial carbon dioxide partial pressure (PaCO_2_, mmHg). **(C)** The ordinate represents peak inspiratory pressure (Ppeak, cmH_2_O). **(D)** The ordinate represents mean airway pressure (Pmean, cmH_2_O). **(E)** The ordinate represents plateau pressure (Pplat, cmH_2_O). **(F)** The ordinate represents dynamic compliance (Cdyn, cmH_2_O).

The changes in gas exchange function and respiratory mechanics between the two groups are detailed in Table 2. After PSM, there were no significant differences between the two groups in all the measured parameters at T1 (*P* > 0.05). At T2, the PCV-VG group exhibited smaller changes in PaCO_2_, Pmean, Ppeak, Pplat, and Cdyn compared to the VCV group, with statistically significant differences observed (*P* < 0.05). By T3, most parameters in both groups had returned to levels close to those at T1, with no significant differences between the groups (*P* > 0.05). However, the PCV-VG group showed a more pronounced recovery in PaCO_2_, with statistically significant differences compared to the VCV group (*P* < 0.05). Figure 2 shows the changes in gas exchange function and respiratory mechanics after PSM. It can be observed that from T1 to T3, PaCO₂, Pmean, Ppeak, and Pplat initially rise and then fall, while PaO₂ and Cdyn first decrease and then increase.

**Table 2:** Changes in gas exchange function and respiratory mechanics at different time.

Variable	Group	T1	T2	T3
PaO_2_ (mmHg)	VCV	143.0 (141.0, 146.0)	87.0 (82.0, 96.0)	153.78 ± 3.67
	PCV-VG	143.0 (139.0, 148.0)	92.0 (89.0, 98.0)	155.22 ± 4.40
	*P*	0.888	0.032	0.133
PaCO_2_ (mmHg)	VCV	41.43 ± 5.17	60.30 ± 3.70	47.62 ± 3.55
	PCV-VG	40.70 ± 3.20	53.22 ± 3.61	43.00 ± 3.50
	*P*	0.468	<0.001	<0.001
Pmean (cmH_2_O)	VCV	7.00 (7.00, 7.00)	12.00 (12.00, 13.00)	7.00 (7.00, 8.00)
	PCV-VG	7.00 (6.00, 8.00)	10.00 (9.00, 10.00)	7.00 (6.00, 8.00)
	*P*	0.591	<0.001	0.084
Ppeak (cmH_2_O)	VCV	16.00 (14.00, 17.00)	22.59 ± 1.79	17.35 ± 1.58
	PCV-VG	16.00 (15.00, 17.00)	20.95 ± 1.67	17.22 ± 1.65
	*P*	0.756	<0.001	0.721
Pplat (cmH_2_O)	VCV	15.00 (14.00, 15.00)	18.78 ± 2.08	15.00 (15.00, 16.00)
	PCV-VG	14.00 (13.00, 15.00)	16.27 ± 1.19	15.00 (14.00, 16.00)
	*P*	0.549	<0.001	0.386
Cdyn (cmH_2_O)	VCV	24.00 (23.00, 24.00)	13.19 ± 1.33	24.14 ± 1.90
	PCV-VG	23.00 (23.00, 24.00)	16.70 ± 1.22	23.73 ± 1.30
	*P*	0.580	<0.001	0.289

**Figure 3:**
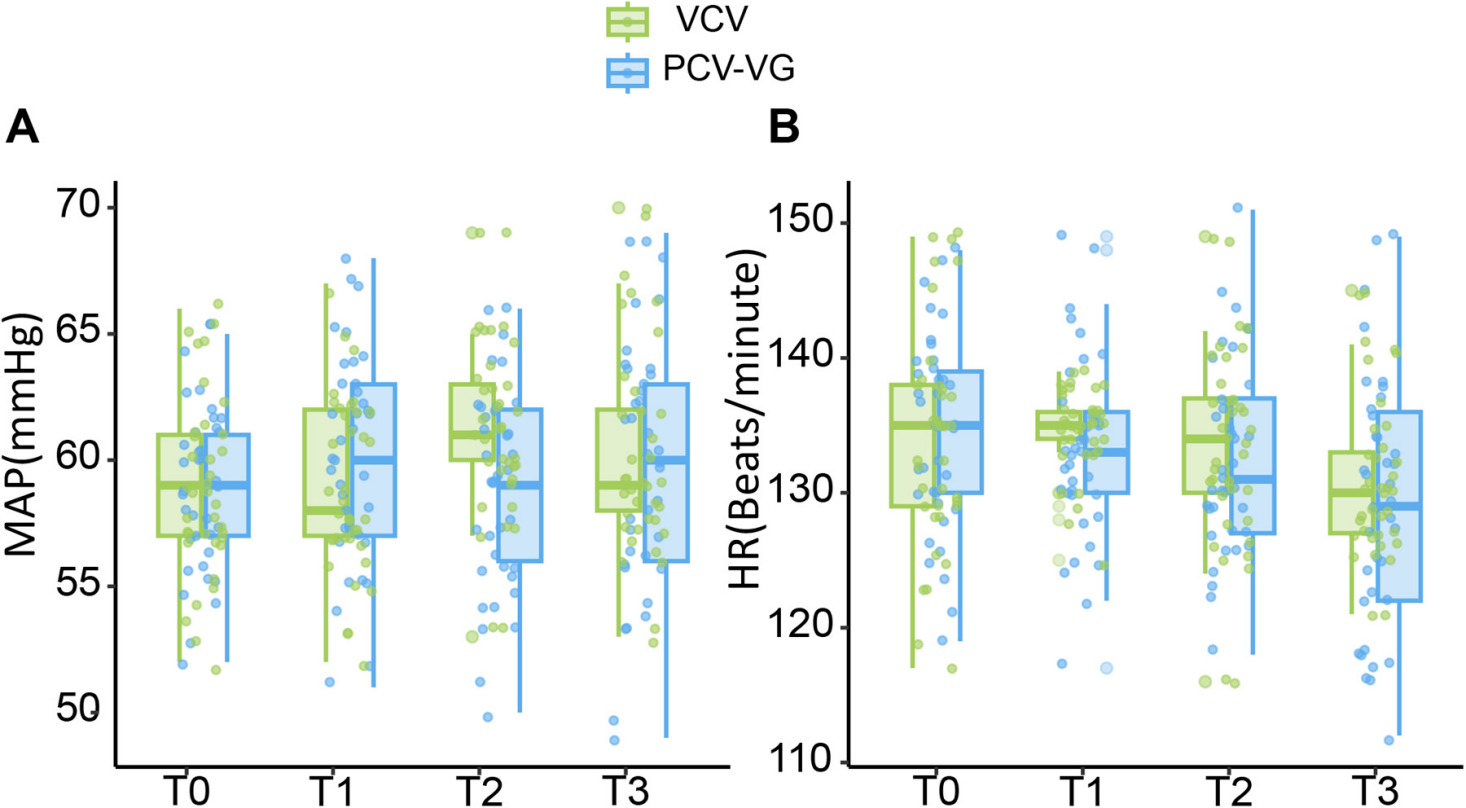
Changes in MAP and HR. T0: before tracheal intubation. **(A)** The ordinate represents mean arterial pressure (MAP, mmHg). **(B)** The ordinate represents heart rate (HR, Beats/minute).

The changes in MAP and HR between the two groups are shown in Table 3. At T0 and T1, there were no significant differences between the two groups in terms of MAP and HR (*P* > 0.05). At T2, the MAP in the PCV-VG group decreased significantly compared to the VCV group, with a statistically significant difference observed (*P* < 0.05). However, there were no significant differences in HR between the two groups (*P* > 0.05). By T3, both MAP and HR in the two groups returned to similar levels, with no significant differences observed (*P* > 0.05). Figure 3 illustrates the changes in MAP and HR after PSM, indicating that from T1 to T3, MAP and HR remained relatively stable.

**Table 3:** Changes in MAP and HR.

Variable	Group	T0	T1	T2	T3
MAP (mmHg)	VCV	58.92 ± 3.51	58.95 ± 3.45	61.27 ± 3.57	60.19 ± 4.21
	PCV-VG	58.92 ± 3.25	60.14 ± 4.15	58.76 ± 4.08	59.81 ± 5.02
	*P*	1.000	0.238	0.006	0.727
HR	VCV	133.84 ± 8.18	135.00 (134.00, 138.00)	133.32 ± 7.42	131.03 ± 6.04
	PCV-VG	134.51 ± 7.14	133.00 (130.00, 136.00)	132.62 ± 7.22	129.19 ± 9.44
	*P*	0.730	0.082	0.670	0.323

Table 4 presents the comparison of postoperative mechanical ventilation duration, ICU length of stay, and overall hospital stay between the two groups. The PCV-VG group had significantly reduced times for mechanical ventilation, ICU stay, and hospital stay compared to the VCV group (*P* < 0.05).

**Table 4:** Comparison of extubation time, ICU and hospital stay duration between the two groups.

Characteristics	VCV	PCV-VG	*P*
Extubation time [median (IQR)], days	3.4 (2.9, 3.6)	3.2 (2.5, 3.2)	<0.001
ICU stay duration [median (IQR)], days	4.8 (4.3, 5.0)	4 (3.0, 5.0)	0.020
Hospital stay duration [median (IQR)], days	16.0 (16.0, 18.0)	11.0 (10.0, 13.0)	<0.001

### Comparison of postoperative complications

There was no significant variations were observed in the incidence of anastomotic leakage, anastomotic stricture, esophageal-tracheal fistula recurrence, reflux esophagitis, and tracheomalacia between the VCV and PCV-VG groups (*P* > 0.05, Table 5).

**Table 5:** Comparison of postoperative complications between the two groups.

Characteristic	VCV	PCV-VG	*P*
Anastomotic leakage	15 (47.5%)	12 (32.4%)	0.469
Anastomotic stricture	5 (13.5%)	3 (8.1%)	0.711
Esophageal-tracheal fistula recurrence	3 (8.1%)	2 (5.4%)	1.000
Reflux esophagitis	6 (16.2%)	4 (10.8%)	0.496
Tracheomalacia	10 (27.0%)	6 (16.2%)	0.167

## Discussion

In our study, we employed PSM to balance the baseline characteristics of the two groups. By comparing the PCV-VG mode and VCV mode, we found that during OLV, the PCV-VG mode demonstrated better gas exchange function and more stable respiratory mechanics compared to the VCV mode. Additionally, the PCV-VG group had significantly shorter extubation time, ICU and hospital stay, indicating the potential advantages of this mode in neonatal thoracoscopic surgery. MAP showed persistent differences between the groups, while HR did not exhibit significant differences before and after matching. Overall, the PCV-VG mode may offer advantages in reducing intraoperative and postoperative complication risks and accelerating postoperative recovery. These benefits are particularly important for the fragile neonatal lungs and may contribute to improved surgical outcomes.

When transitioning from bilateral to OLV, the reduction in effective gas exchange area leads to diminished oxygenation and carbon dioxide clearance efficiency. Clinically, increasing the fraction of inspired oxygen (FiO₂), adjusting tidal volume (Vt), respiratory rate, and optimizing positive end-expiratory pressure (PEEP) are common strategies to maintain blood gas homeostasis (11–13). However, conventional VCV, with its fixed tidal volume settings, predisposes to abnormal elevations in airway pressures. Ppeak, Pmean, and Pplat represent the peak airway pressure during a respiratory cycle, the mean airway pressure over multiple cycles, and the approximate average alveolar pressure, respectively. Elevations in these parameters typically indicate increased airway resistance or decreased Cdyn, thereby escalating the risk of lung injury (14). Our study observed that compared with traditional VCV, PCV-VG dynamically adjusts airway pressure by real-time monitoring of Cdyn, significantly reducing these pressure indices while ensuring target tidal volume delivery. A prospective study by Yang et al. (15) in neonates aged 1–4 days further validated the advantages of PCV-VG in extremely early neonates: during OLV, Ppeak was approximately 7.25% lower, Cdyn increased by approximately 26%, and PaO₂ and pH levels were higher, while PaCO₂ was lower in the PCV-VG group, indicating superior gas exchange and stronger protective effects on immature lung tissue (15).

PCV-VG mitigates the pressure surges caused by declining Cdyn in VCV through its decelerating flow pattern and automatic pressure compensation. Studies in infants (1 month to 3 years) and toddlers (2–12 months) have demonstrated consistent cross-age benefits of PCV-VG: in pediatric patients aged 1–3 years, this mode significantly improves oxygenation (higher PaO₂) by reducing Ppeak, Pmean, and enhancing Cdyn during OLV (16). In 2–12-month-old children undergoing thoracoscopic OLV, PCV-VG notably increases Cdyn and reduces both Ppeak and Pmean (17). These findings, complementary to the neonatal studies [current study and (15)], demonstrate the protective effects of PCV-VG across different stages of lung development. Notably, in the context of immature neonatal lungs, its dynamic pressure regulation mechanism holds particular clinical value.

The VCV mode controls ventilation by setting a fixed Vt, ensuring a constant volume of gas delivered to the lungs with each breath. To maintain this fixed tidal volume, the VCV mode may lead to increased airway pressures, particularly when lung compliance decreases or airway resistance increases, thereby raising the risk of volume trauma and barotrauma (18). In contrast, the PCV-VG mode combines the advantages of pressure control and volume control. By setting a target tidal volume, this mode automatically adjusts the pressure to ensure a consistent ventilation volume. When lung compliance or airway resistance changes, PCV-VG adjusts the pressure to maintain the preset tidal volume, thereby reducing the risk of inadequate or excessive ventilation (8). This automatic adjustment feature is particularly crucial in neonatal thoracoscopic surgery.

PaO₂ and PaCO₂ are critical indicators of respiratory function and play a key role in assessing ventilation effectiveness and guiding treatment decisions. During surgery, factors such as significant CO₂ absorption from the thoracic cavity, increased pulmonary shunting, and mechanical compression can lead to impaired pulmonary gas exchange in neonates (19). In this complex physiological environment, the dynamic regulation capabilities of PCV-VG may be more beneficial in maintaining stable ventilation and oxygenation. Our study shows that PaCO₂ increased in both groups of neonates after 30 min of OLV. Despite strict control of thoracic CO₂ infusion rates and pressures during surgery, and the use of intermittent manual ventilation to promote CO₂ expulsion, CO₂ accumulation was still observed. Factors such as CO₂ absorption during surgery, lung compression, and pulmonary shunting may further exacerbate gas exchange difficulties, which could contribute to elevated PaCO₂ levels (20). The neonate group using PCV-VG showed PaCO₂ levels closer to the normal range, suggesting that PCV-VG may help optimize pulmonary gas exchange during OLV. Although PCV-VG automatically adjusts pressure according to the set tidal volume, excessively high target tidal volumes could lead to excessive pressure, increasing the risk of barotrauma (21). It is important to note that PaO₂ and PaCO₂ not only play a critical role in maintaining intraoperative ventilation status but also directly impact postoperative recovery and hospital stay. Our study indicates that the extubation time, ICU and hospital stay were significantly reduced in the PCV-VG group. Liu et al. (22) further support this finding, noting that lower PaO₂ and higher PaCO₂ are significantly associated with prolonged hospital stays. This underscores the importance of appropriately setting and dynamically adjusting ventilation parameters, such as tidal volume and pressure, in this vulnerable population to minimize CO₂ accumulation and optimize postoperative recovery.

In terms of postoperative complication rates, whether or not PSM was performed, there were no significant differences between the VCV and PCV-VG groups in the incidence of anastomotic leakage, anastomotic stenosis, recurrence of tracheoesophageal fistula, reflux esophagitis, and tracheomalacia. Postoperative complications may be primarily influenced by surgical technique and the experience of the surgeon, with the impact of ventilation mode on these complications likely being limited. Additionally, factors such as the underlying condition of the neonate, nutritional status, and immune function may also affect the occurrence of complications. The study might not have captured differences in long-term complications. Although ventilation mode may not directly affect the incidence of complications, it could indirectly influence prognosis by improving intraoperative conditions. The PCV-VG mode enhanced intraoperative respiratory mechanics and gas exchange, potentially creating a better surgical environment, which could indirectly impact surgical quality and outcomes. Given the extended recovery time post-surgery, our research team is currently conducting ongoing assessments of the long-term neurological development of these neonates. These assessments are still in progress, and final results have not yet been obtained.

This study has a few limitations. Firstly, it primarily focused on the short-term incidence of complications, potentially missing differences in long-term complications. Given the rarity of esophageal atresia, the sample size may have been insufficient to detect subtle variations in complication rates. Furthermore, the study only assessed the complication incidence without evaluating the severity and duration. Regarding ventilation mode allocation, the non-randomized design—with VCV predominantly used in early cases and PCV-VG adopted later as equipment and clinical experience evolved—introduces potential historical bias. Perioperative management improvements during the study period could confound the true effects of the ventilation mode itself. While PSM was employed to balance baseline characteristics, residual confounding from time-related factors (e.g., advancements in anesthetic techniques) cannot be fully eliminated. Lastly, the study may not have fully controlled for other variables influencing complication occurrence, such as neonate's underlying condition and nutritional status. For future research, long-term follow-up is needed to assess how different ventilation modes affect extended neonatal prognosis. Multi-center collaborations should be prioritized to expand sample size and enhance statistical power. Multi-center collaborations should be prioritized to expand sample size and enhance statistical power. Complications must be evaluated comprehensively, including their incidence, severity, and duration. Additionally, exploring individualized treatment protocols and optimizing perioperative management strategies are critical next steps.

## Conclusion

Our retrospective analysis indicates that PCV-VG during thoracoscopic repair of esophageal atresia may correlate with improved intraoperative respiratory mechanics and shorter mechanical ventilation duration/ICU stay vs. VCV. However, the non-randomized design and potential confounding factors limit definitive conclusions about ventilator superiority. Notably, postoperative complication rates were comparable between groups, underscoring the need for further research to clarify long-term outcomes.

## Data Availability

The raw data supporting the conclusions of this article will be made available by the authors, without undue reservation.
